# Drought adaptation of stay-green sorghum is associated with canopy development, leaf anatomy, root growth, and water uptake

**DOI:** 10.1093/jxb/eru232

**Published:** 2014-06-13

**Authors:** Andrew K. Borrell, John E. Mullet, Barbara George-Jaeggli, Erik J. van Oosterom, Graeme L. Hammer, Patricia E. Klein, David R. Jordan

**Affiliations:** ^1^University of Queensland, Queensland Alliance for Agriculture and Food Innovation (QAAFI), Hermitage Research Facility, Warwick, QLD 4370, Australia; ^2^Department of Biochemistry and Biophysics, Texas A&M University, College Station, TX 77843, USA; ^3^Department of Agriculture, Fisheries and Forestry Queensland (DAFFQ), Hermitage Research Facility, Warwick, QLD 4370, Australia; ^4^University of Queensland, QAAFI, Brisbane, QLD 4072, Australia; ^5^Department of Horticultural Sciences, Texas A&M University, College Station, TX 77843, USA

**Keywords:** Canopy development, crop water use, drought adaptation, leaf anatomy, root architecture, sorghum, stay-green.

## Abstract

Stay-green sorghum plants exhibit greener leaves and stems during the grain-filling period under water-limited conditions compared with their senescent counterparts, resulting in increased grain yield, grain mass, and lodging resistance. Stay-green has been mapped to a number of key chromosomal regions, including *Stg1*, *Stg2*, *Stg3*, and *Stg4*, but the functions of these individual quantitative trait loci (QTLs) remain unclear. The objective of this study was to show how positive effects of *Stg* QTLs on grain yield under drought can be explained as emergent consequences of their effects on temporal and spatial water-use patterns that result from changes in leaf-area dynamics. A set of four *Stg* near-isogenic lines (NILs) and their recurrent parent were grown in a range of field and semicontrolled experiments in southeast Queensland, Australia. These studies showed that the four *Stg* QTLs regulate canopy size by: (1) reducing tillering via increased size of lower leaves, (2) constraining the size of the upper leaves; and (3) in some cases, decreasing the number of leaves per culm. In addition, they variously affect leaf anatomy and root growth. The multiple pathways by which *Stg* QTLs modulate canopy development can result in considerable developmental plasticity. The reduction in canopy size associated with *Stg* QTLs reduced pre-flowering water demand, thereby increasing water availability during grain filling and, ultimately, grain yield. The generic physiological mechanisms underlying the stay-green trait suggest that similar *Stg* QTLs could enhance post-anthesis drought adaptation in other major cereals such as maize, wheat, and rice.

## Summary

The positive effects of stay-green quantitative trait loci on grain yield of sorghum under post-anthesis drought are emergent consequences of their effects on water-use patterns, resulting from changes in pre-anthesis canopy size.

## Introduction

The Earth is a water-scarce planet. Feeding more people with less water is a major challenge facing humanity (Foley *et al.*, 2011), requiring crops that are highly adapted to dry environments. One such crop, sorghum, evolved in Africa after splitting with rice 50–70 million years ago (Wolfe *et al.*, 1989) and is an important global crop grown for food, feed, fibre, and fuel (Paterson *et al*., 2009a, 2009b). It is, therefore, a repository of drought adaptation mechanisms. Sorghum is a staple in the semiarid environments of sub-Saharan Africa and central-western India, where people require stable food production. While the global population will increase from about 7 billion to 9 billion by 2050, most of the increase will occur in sub-Saharan Africa, where population growth is among the highest in the world (Haub, 2013), increasing the risk of food insecurity in this region (United Nations Development Programme, 2012). Plant traits such as semidwarfism and enhanced responsiveness to N fertilizer increased food production in the so-called Green Revolution in the 1960s and 1970s (Khush, 2001). Now, a new set of plant traits is needed to further increase crop yield in a Blue Revolution (Pennisi, 2008), making plants resilient to the challenges of a water-scarce planet where climate change and global warming threaten food supplies (Vidal, 2013).

**Fig. 1.**
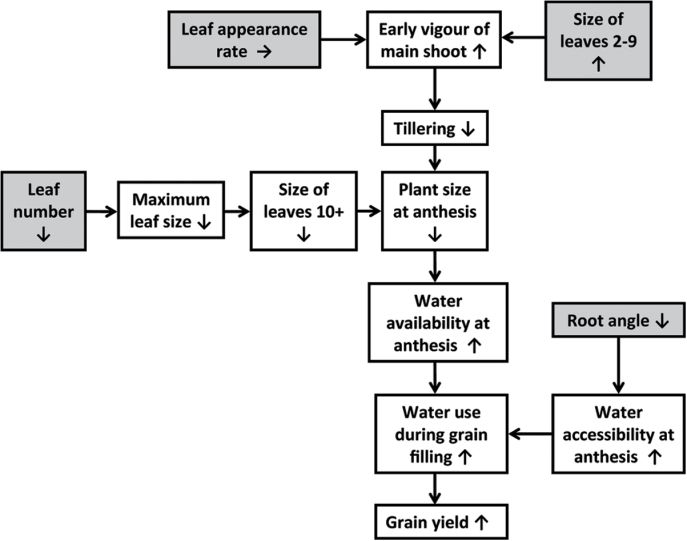
Flowchart of crop physiological processes that determine plant size and crop water use at anthesis, with consequences for water uptake during grain filling. Grey boxes indicate traits that are directly affected by *Stg* QTLs and white boxes indicate traits for which the effect is an emergent consequence of the effect on the grey box. Up arrows indicate increase; down arrows indicate decrease; side arrows indicate no change.

Stay-green is an integrated drought-adaptation trait in sorghum. Delayed leaf senescence during grain filling is an emergent consequence of dynamics occurring earlier in crop growth (Fig. 1) and is largely due to an improved balance between the supply and demand of water, as well as the efficiency with which the crop converts water to biomass and grain yield (Borrell *et al.*, 2009; Jordan *et al.*, 2012). On the supply side, crop water use during grain filling can be enhanced by increasing water availability at anthesis and/or increasing water accessibility during grain filling (van Oosterom *et al.*, 2011). On the demand side, crop water use can be reduced by decreasing leaf area and/or transpiration per unit leaf area. Leaf area can be constrained by reducing tillering (Kim *et al.*, 2010), leaf number per culm, and/or individual leaf size (Borrell *et al.*, 2000a). Transpiration per unit leaf area can be limited by stomatal density or aperture, timing of stomatal opening, and hydraulic factors. This paper highlights how stay-green (*Stg*) quantitative trait loci (QTLs) modify physiological mechanisms affecting both the supply and demand of water to increase drought adaptation.

There are multiple ways for a plant to remain green (Thomas and Howarth, 2000). A stay-green phenotype may arise if the onset of senescence is delayed (type A), the rate of senescence is reduced (type B), chlorophyll is retained but photosynthesis declines (type C), greenness is retained due to rapid death at harvest (type D), or the phenotype is greener to begin with (type E). These classifications indicate that stay-green may be functional or cosmetic. Functional stay-green is characterized by the maintenance of leaf photosynthesis during grain filling (types A, B, and E), while cosmetic stay-green occurs when photosynthetic capacity is disconnected from leaf greenness (types C and D). Enhanced crop productivity in water-limited environments is dependent on functional stay-green. However, not all functional stay-green is necessarily productive. For example, low sink demand relative to source, created by a small panicle or low grain number, will generate a stay-green phenotype since there is little demand for the crop to translocate carbon and nitrogen from leaves to grain (Henzell and Gillieron, 1973; Rosenow *et al.*, 1983). Therefore, selection for both stay-green and grain yield should be undertaken simultaneously in plant breeding programmes to ensure that delayed senescence is not due to low sink demand.

Delayed leaf senescence can be examined at the cell, leaf. or whole-plant levels (Borrell *et al.*, 2001). While analysis at each of these levels is helpful in understanding the overall stay-green phenomenon, it has remained a challenge to link molecular processes to whole-plant physiology and, ultimately, to crop production. To some extent, recent reviews have attempted to integrate knowledge on drought adaptation and senescence across scales from molecular to whole-plant (Mir *et al.*, 2012; Gregersen *et al.*, 2013). However, the link to whole-plant physiology remains the most tenuous. For example, many recent publications on plant senescence are at the molecular level focusing on *Arabidopsis* (Jing and Nam, 2012; Li *et al.*, 2012; Wu *et al.*, 2012; Kim *et al.*, 2013), wheat (Tian *et al.*, 2013), and rice (Jan *et al.*, 2013). At a general level, phenotyping for drought tolerance in the genomics era is beginning to be addressed (Tuberosa, 2012), but such linkages need to be explored more specifically in relation to leaf senescence. Identifying the genes underpinning the key stay-green QTLs in cereals is underway (Harris *et al.*, 2007; Borrell *et al.*, 2009), and this knowledge, combined with physiological understanding, should help to bridge the molecular to whole-plant gap.

This paper integrates results from a number of experiments that were conducted to elucidate the physiological mechanisms underpinning the BTx642 (formerly B35) source of stay-green in sorghum. Critical to this research was the development of specific genetic populations (e.g. recombinant inbred lines, RILs; near-isogenic lines, NILs; fine-mapping populations) to enable QTL mapping (Crasta *et al.*, 1999; Tuinstra *et al*., 1996, 1997, 1998; Boffa *et al.*, 2000; Subudhi *et al.*, 2000; Tao *et al.*, 2000; Xu *et al.*, 2000), physiological dissection (Harris *et al.*, 2007), and fine mapping for gene discovery (Borrell *et al.*, 2009). In particular, the development of NILs containing single introgressions of the *Stg1*, *Stg2*, *Stg3*, and *Stg4* QTLs from BTx642 in a RTx7000 background was crucial to understanding the contribution of each QTL alone to drought adaptation in sorghum (Harris *et al.*, 2007).

The objective of this paper was to show how the positive effects of *Stg* QTLs on grain yield under drought can be explained as a consequence of their effects on temporal and spatial water-use patterns that result from changes in leaf-area dynamics.

## Materials and methods

### Construction of RTx7000 NILs containing BTx642 DNA from the stay-green loci

The construction of RTx7000 NILs containing one or more of the *Stg* loci from BTx642 has been described in detail by Harris *et al.* (2007). In brief, the BTx642 source of stay-green is derived from IS12555, a durra landrace from Ethiopia (Borrell *et al.*, 2000a). F1 plants of a cross between BTx642 and RTx7000 were backcrossed to RTx7000 either four (BC4, 6000 NIL series) or six (BC6, 2000 NIL series) times and progenies from each backcross were screened for the presence of *Stg* loci (Harris *et al.*, 2007). Selected plants were then selfed to create BC4F2–4 or BC6F2–4 lines. Although most experiments contained many genotypes, only data on four RTx7000 NILs, each containing only either the *Stg1* NIL (6078-1), *Stg2* NIL (2219-3), *Stg3* NIL (2290-19), or Stg4 NIL (6085–9), plus the two parents (RTx7000 and BTx642) are reported here. The size and location of BTx642 DNA introgressions in the RTx7000 NILs, including the four examined in detail in this paper, have been reported by Harris *et al.* (2007).

### Field experiments

Details of most field experiments are given in Borrell *et al.* (2014). In summary, four field experiments were conducted across two locations in Australia’s northeastern grain belt: Biloela (BIL: 24° 24′ S, 150° 30′ E; elevation 175 m) in central Queensland, and Warwick (WAR: 28° 12′ S, 152° 06′ E; elevation 480 m) in southeastern Queensland. Experiment names are constructed by a combination of location (BIL, WAR), year (e.g. 09 for 2009), and how the experiments were conducted (e.g. FLD for field). Field experiments were used to assess the effects of *Stg* QTLs on phenology, canopy development, crop water use, and grain yield (Table 1).

**Table 1.** A summary of key parameters evaluated in this paper for a range of field, lysimetry and pot experimentsGLAA, green leaf area at anthesis; GN, grain number; GNPP, grain number per panicle; GS, grain size; GY, grain yield; HD, high-density treatment; HW, high-water treatment; LD, low-density treatment; LS, leaf size; LW, low-water treatment; RHI, root harvest index; RM, root mass; SI, stomatal index (abaxial); T, transpiration; T/LA, transpiration per unit leaf area; TL, tillering; VPD, vapour pressure deficit.

Experiment	Sowing date	Water treatment	VPD	Plant density	Genotypes evaluated	Canopy dynamics	Root dynamics	Water use	Plant water status	Grain yield and components
BIL02FLD	18 February 2002	LW, HW	Ambient	Standard	BTx642, RTx7000, *Stg1*, *Stg2*, *Stg3*, *Stg4*	GLAA, TL	–	–	–	–
WAR04FLD	11 December 2003	LW	Ambient	HD, LD	RTx7000, *Stg1*, *Stg2*, *Stg3*	GLAA, TL, LS	–	–	–	GY, GN, GNPP, GS
WAR05FLD	21 January 2005	LW	Ambient	HD, LD	RTx7000, *Stg1*, *Stg2*, *Stg3*, *Stg4*	GLAA, TL, LS	–	T	T/LA	GY, GN, GS
WAR05FLD	7 January 2005	HW	Ambient	HD, LD	RTx7000, *Stg1*, *Stg2*, *Stg3*, *Stg4*	GLAA, TL, LS	–	–	SI	GY
WAR06FLD	25 January 2006	LW, HW	Ambient	HD, LD	RTx7000, *Stg1*	GLAA, TL, LS	–	–	–	–
WAR06LYS	25 February 2006	HW	Low	LD	RTx7000, *Stg1*, *Stg2*, *Stg3*, *Stg4*	GLAA, TL, LS	–	T	T/LA	–
WAR07LYS	22 February 2007	HW	High	LD	RTx7000, *Stg1*, *Stg2*, *Stg3*, *Stg4*	GLAA, TL, LS	–	T	T/LA	–
WAR08POT	7 December 2007	HW	Ambient	LD	*Stg4* fine-mapping population	GLAA, TL, LS	RM, RHI	–	–	–
WAR10POT	10 December 2009	HW	Ambient	LD	RTx7000, *Stg1*	GLAA, TL, LS	–	–	–	–

The first experiment was sown at Biloela on 18 February 2002 (BIL02FLD) on a soil with a dark sandy clay loam A horizon over a brown silty clay B horizon (Northcote, 1979). The experiment included a well-watered (high water, HW) and post-flowering deficit (low water, LW) treatment and was laid out as a split plot with irrigation treatments as main plots, genotypes as subplots, and three replicates. Main plots were 54×19 m, subplots consisted of three rows of 9 m length with 0.9 m row spacing. Data were collected only from the centre row of each plot. Irrigation, fertilizer application, and insect and weed control are outlined in Borrell *et al.* (2014).

Three experiments were conducted at Warwick on a cracking and weakly self-mulching brownish-black clay (McKeown, 1978; Northcote, 1979). The first experiment (WAR04FLD) was sown on 11 December 2003 and emerged 5 days later (Table 1). The second experiment (WAR05FLD) was sown on 7 January 2005 (HW) and 21 January 2005 (LW) and emerged after 8 (HW) and 4 (LW) days. The third experiment (WAR06FLD) was sown on 25 January 2006 for both the HW and LW treatment and emerged 4 days later. The experiments were conducted under nonlimiting nutrient conditions and were planted on full profiles of subsoil moisture (Borrell *et al.*, 2014). Since the LW treatment depended on rainfall exclusion via rain-out shelters, water treatments could not be randomly allocated within replicates and the HW treatment was a separate block adjacent to the rain-out shelter (LW treatment). Each treatment block was laid out as a split plot with density (HD and LD, 20 and 10 plants m^–2^ respectively) as main plots, genotypes as subplots, and four replications. Hence, four treatments with increasing levels of water deficit were created, ranging from HWLD (least stressed) to HWHD, LWLD, and LWHD (most stressed). Plots consisted of four rows of 3 m length with 0.5 m row spacing.

### Semicontrolled environment experiments

Experiments in semicontrolled environments were conducted at Warwick in ventilated, plastic-covered growth tunnels that excluded rainfall and transmitted approximately 70% of the incident solar radiation (Borrell *et al.*, 2014). Experiments consisted of individual plants grown in lysimeters (LYS) or small pots (POT). The LYS were made from cylindrical polyvinyl chloride tubes, 300mm diameter and 750mm high. Each was filled with a 3:1:1 mix of alluvial clay soil, loam, and feedlot manure, and 30g Osmocote Plus (16% N, 3.5% P, 10% K plus trace elements; Scotts, Baulkham Hills, Australia) was added to each lysimeter. Experiment WAR06LYS was sown on 25 February 2006 and WAR07LYS on 22 February 2007. Both experiments were laid out as randomized complete block designs with either seven (WAR06LYS) or four replications (WAR07LYS). Plants were well watered and harvested soon after anthesis. Lysimeters are sealed pots that provide detailed data on plant water use, and because their volume is sufficiently large to minimize effects on plant growth (Yang *et al.*, 2010), they complement data from field experiments. The impact of various *Stg* QTLs on parameters linking leaf area and transpiration under low (WAR06LYS, 1.10 kPa) and high (WAR07LYS, 1.54 kPa) vapour pressure deficit conditions were evaluated (Table 1).

Pot experiments were conducted at Warwick during the summers of 2007/2008 (WAR08POT) and 2009/2010 (WAR10POT) (Table 1). The WAR08POT experiment was conducted in 7-l planter bags, filled with pure alluvial clay, which were placed on capillary mats to prevent water stress. Plants were well watered and fertilized until harvest when 11 leaves had fully expanded (Borrell *et al.*, 2014). A similar process was followed for WAR10POT, except that larger 19-l pots were used and plants were harvested at anthesis. Both experiments were well fertilized and were laid out as a randomized block design with either four (WAR08POT) or 20 (WAR10POT) replications. Conditions in the growth tunnel provided an environment for phenotyping of mapping populations in POT experiments with less error variance than FLD experiments.

### Phenology and leaf-area development

Emergence was defined as the date when 50% of the plants in each experiment had emerged from the soil and anthesis when, on average, 50% of the anthers had extruded from the main shoot panicle of four tagged plants per plot in FLD studies or from each plant in LYS and POT studies. Physiological maturity was defined as the date when basal grains in 90% of the same tagged plants in FLD studies attained a black layer (Eastin *et al.*, 1973).

In field experiments, total and fertile tiller number per plant were recorded on all plants in one of the central rows of each plot, excluding plants near the end of the rows. The area of all fully expanded leaves on all axes was measured with a planimeter (Delta-T DIAS image analysis system, Cambridge, UK) on two tagged plants per plot at three harvest times, corresponding with the expansion of the 6th, 12th, and flag leaves. In the LYS and POT experiments, tiller number and the area of all fully expanded leaves on all axes were measured for each plant (Table 1). Additional details on measurements are given in Borrell *et al.* (2014).

### Biomass and grain yield sampling

Biomass samples were taken at anthesis and maturity in the three field experiments at Warwick by cutting plants at ground level and dividing them into green and senesced leaves, stems (including leaf sheaths), and panicles (if present) for main shoots and all tillers combined. Green leaf area of the whole sample was obtained with a planimeter. Samples were dried at 80 °C for at least 2 days before obtaining dry mass. After threshing of dried panicles, grain yield and 100 grain mass were measured and grain number was derived.

### Crop water use

In WAR05FLD, soil water content was measured by neutron moderation (model 503 DR Hydroprobe, CPN International, CA, USA). One access tube per plot was sited at the mid inter-row position. Readings were taken weekly at 20-cm depth intervals to a depth of 1.8 m throughout crop growth and were converted to soil water content using a calibration equation (Borrell *et al.*, 2014). Soil water content in the 0–20cm layer was determined gravimetrically by taking a core within a radius of 1 m from the access tube. For each measurement date, total soil water content was taken as the sum of individual depth intervals and temporal patterns of cumulative soil water content were developed for each plot.

In WAR06LYS and WAR07LYS, the sealed LYS were weighed weekly and any change in weight was recorded as the amount of transpiration by the plant. LYS were then rewatered to their starting weight and plant water use was calculated as the sum of the amount of water added (Borrell *et al.*, 2014). Water loss from blank sealed pots (no plant) was measured weekly and found to be negligible. Plants were harvested soon after anthesis.

### Stomatal index

Stomatal density depends on the epidermal cell size, the leaf position, and environmental factors that affect epidermal cell expansion (Tichá, 1982; Royer, 2001). As a consequence, stomatal density can be highly variable, even over the surface of a single leaf (Woodward, 1993). To standardize for the effects of epidermal cell expansion, stomatal index (SI) was defined by Salisbury (1927) as the number of stomata per unit leaf area, expressed as a percentage of the combined number of stomata, epidermal cells, and hair bases per unit leaf area. The SI is thus a measure of the rate of stomatal initiation that accounts for variability due to epidermal cell size.

Measurements of SI were taken on four replicate plants of RTx7000 and the four *Stg* NILs from the HD and LD treatments in the irrigated control of WAR05FLD. Samples were taken from the abaxial surface of four leaf positions within each plant: leaf 7 (L7), L10, L13, and L16, which were sampled at 24, 32, 42, and 54 days after emergence, respectively. One 0.5-cm^2^ section, located midway between the midrib and leaf margin in the widest part of the leaf where veins were running parallel to each other, was removed from each leaf using a scalpel blade. Leaf sections were cuticularized by boiling in a 6:1 solution of 30% hydrogen peroxide and 100% acetic acid, as reported by Carr and Carr (1991) for *Eucalyptus* leaves. Once the cellular and vascular material was digested, the remaining abaxial cuticle with adhering epidermal layer was washed in distilled water and mounted unstained in glycerine jelly onto glass microscope slides. Slides were examined using a differential phase contrast light microscope (Olympus BX51, Olympus, Tokyo, Japan) with a digital camera mounted to the eyepiece and connected to a colour monitor. Statistical analysis of data was carried out in GraphPad Prism (GraphPad Software, OK, USA).

### Transpiration per unit leaf area

In WAR05FLD, where cumulative crop water use was determined by the neutron moderation method, transpiration per unit leaf area at anthesis was calculated by dividing cumulative crop water use (mm) by total green leaf area (m^2^ m^–2^). It was assumed that transpiration was essentially equal to crop water use, since the remaining water balance components (evaporation from the dry soil surface prior to anthesis, run-off, seepage, and drainage) were considered to be negligible, particularly under the terminal drought conditions in the rain-out shelter.

### Statistical analyses

For the analyses across experiments, each of the four water × density treatments in WAR05FLD was considered an individual experiment. In all experiments, a linear mixed model was fitted for each trait using the ASReml program (Butler *et al.*, 2009) with the R software package (R Development Core Team, 2012). The fitted model contained fixed effects for experiment, genotype, and genotype × experiment interaction, and random effects for replicates and residuals for each experiment. Predicted values for genotype × experiment were calculated using the R function predict.asreml. Significance levels of the fixed effects were determined using a chi-squared Wald test.

## Results and discussion

### *Stg* loci increase grain yield under water-limiting conditions with minimal yield cost in water-sufficient environments

**Fig. 2.**
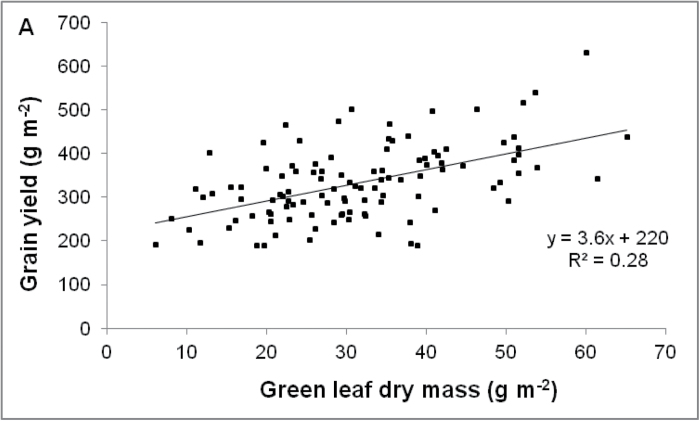

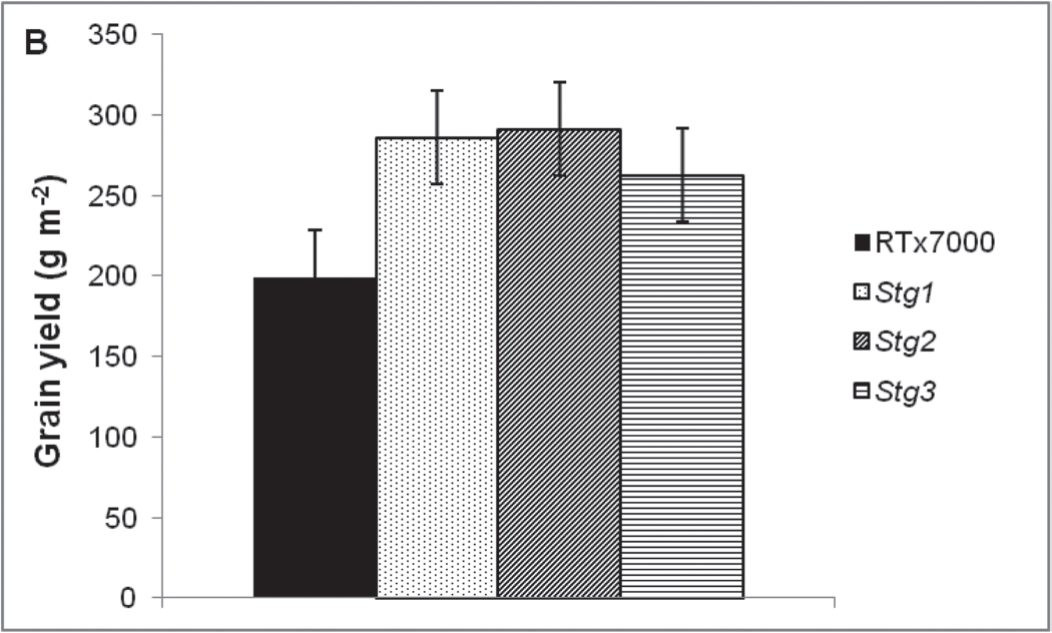

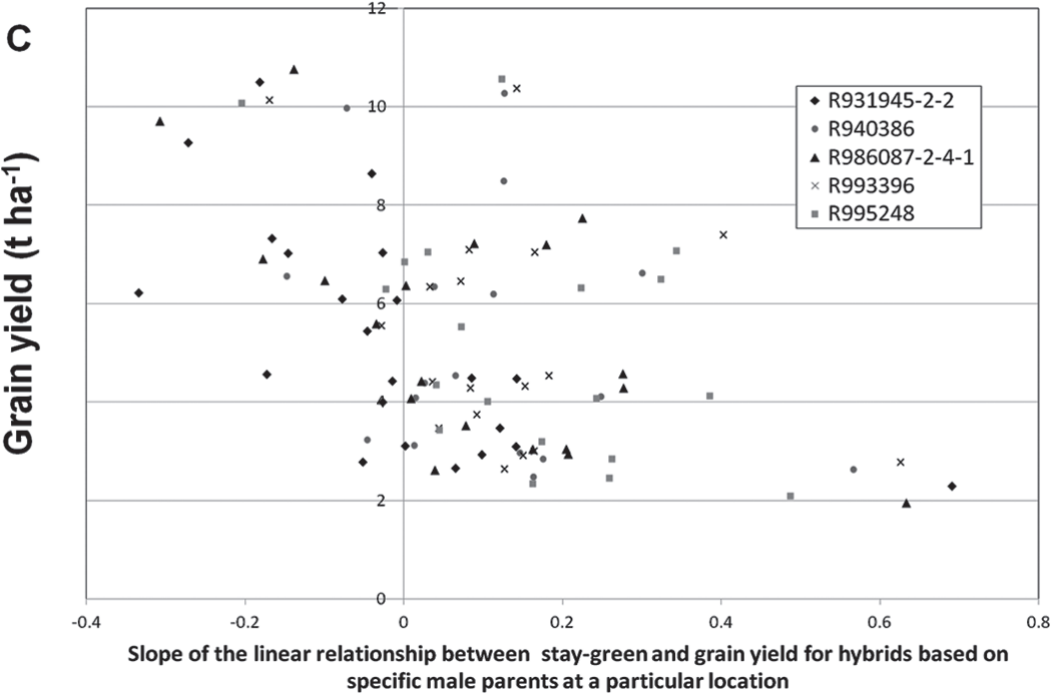
The stay-green trait enhances grain yield in sorghum under post-anthesis drought. (A) Relationship between green leaf dry mass at 25 days after anthesis and grain yield in a set of 160 recombinant inbred lines from the cross between BQL39 (senescent) and BQL41 (stay-green), grown during the post-rainy season at Patancheru, India. (B) Grain yield of *Stg* NILs is enhanced relative to RTx7000 under post-anthesis drought in a rain-out shelter study (WAR04FLD); data are mean±2SE. (C) Grain yield of hybrids from a particular male parent at a particular location plotted against the slope of the relationship between stay-green and yield for the same set of hybrids at that location (Jordan *et al.*, 2012; reprinted by permission, ASA, CSSA, SSSA).

The stay-green trait is positively correlated with sorghum grain yield in field conditions under terminal drought (Borrell *et al*., 1999, 2000b; Jordan *et al*., 2003, 2012). Establishing this correlation is important because sorghum breeders were initially concerned that leaves may remain green simply because of a small sink demand, indicating that stay-green may be correlated with low grain yield (Henzell and Gillieron, 1973; Duncan *et al.*, 1981; Rosenow *et al.*, 1983; Tangpremsri, 1989). One of the most convincing earlier pieces of evidence came from a trial at ICRISAT, India, where a strong positive relationship between green leaf dry mass at 25 days after anthesis and grain yield in a set of 160 RILs (BQL39 (senescent) × BQL41 (stay-green)) was demonstrated for field-grown sorghum in the post-rainy season (Fig. 2A; Borrell *et al.*, 1999).

Although a positive correlation between stay-green and yield has been demonstrated in these earlier studies, the physiological and molecular basis of the stay-green trait still remains unclear. Gaining such insights requires the use of NILs (Table 1). Individual *Stg* NILs consistently yielded more grain than RTx7000 under drought stress in WAR04FLD and WAR05FLD. This difference was significant (*P*<0.05) in all but three of the 14 treatment × *Stg* QTL combinations (Borrell *et al.*, 2014). For example under severe terminal drought stress in a rain-out shelter at WAR04FLD, *Stg1*, *Stg2*, and *Stg3* (*Stg4* was not evaluated) yielded significantly (*P*<0.01) more grain than RTx7000 (Fig. 2B), with the yield benefit ranging from 24% (*Stg3*) to 31% and 32% (*Stg1* and *Stg2*, respectively). Similarly in WAR05FLD, the yield benefit ranged from 12–17% for *Stg2*, *Stg3*, and *Stg4* to 36% for *Stg1* (Borrell *et al.*, 2014).

**Fig. 3.**
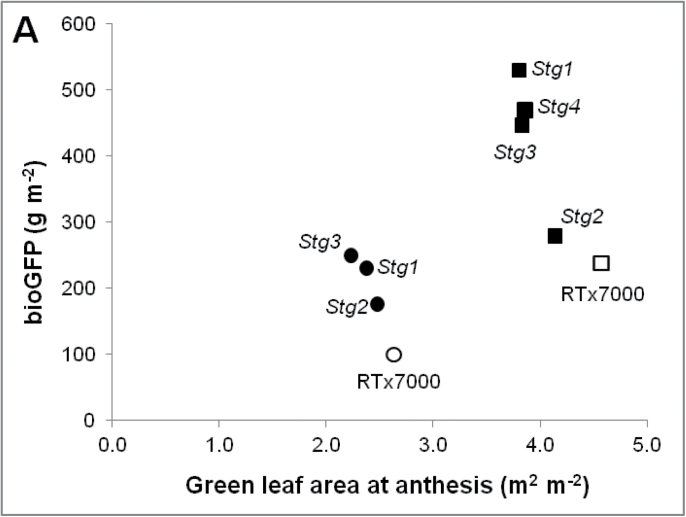

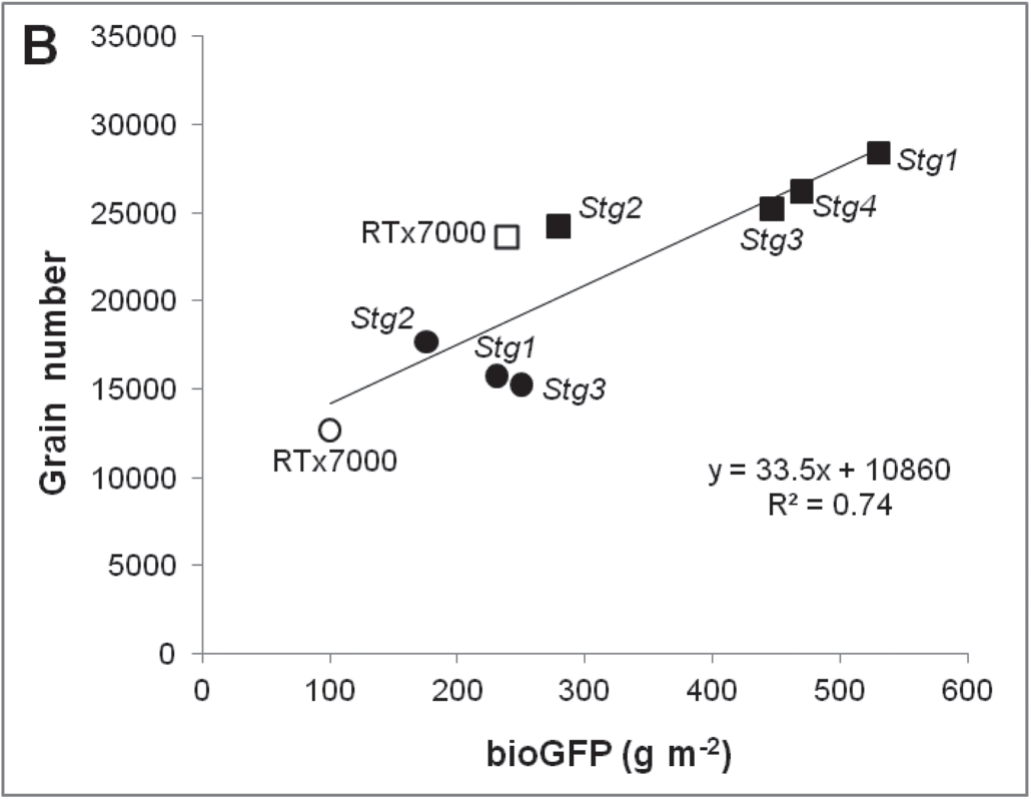

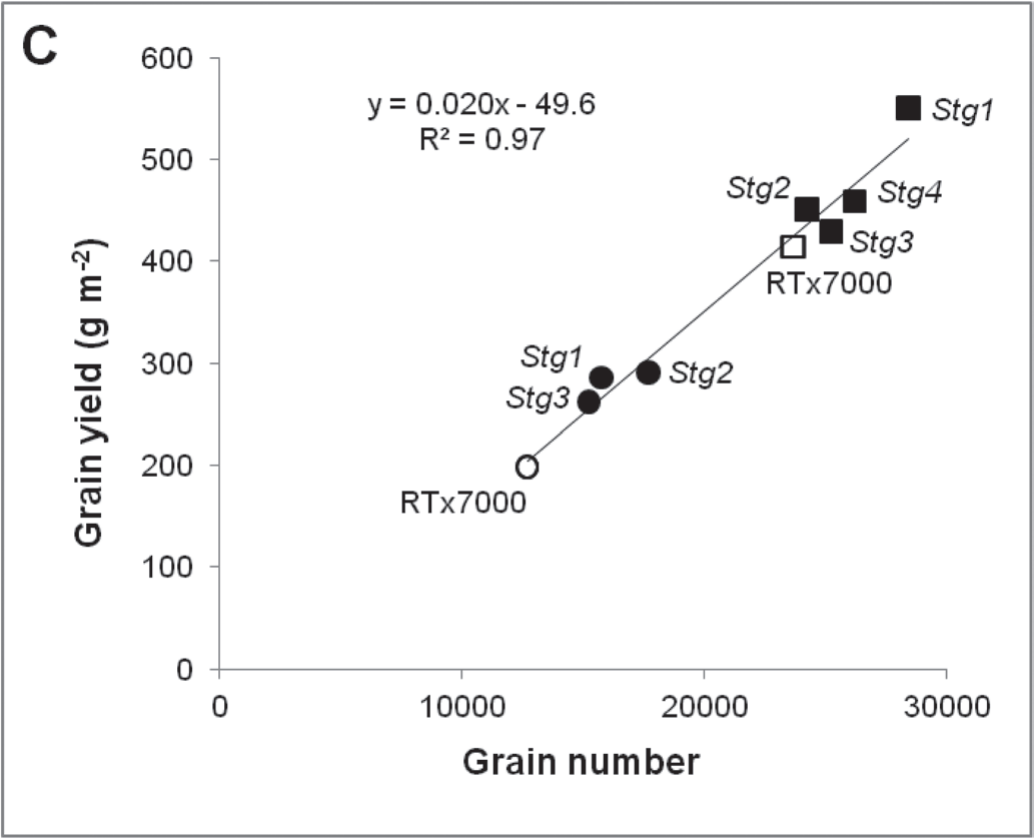
*Stg* QTLs reduce canopy size at anthesis and increase grain yield. (A) Green leaf area at anthesis versus change in biomass during grain filling (bioGFP). (B) Biomass accumulation during grain filling versus grain number. (C) Grain number versus grain yield for RTx7000 (open circle) and four *Stg* QTL NILs (filled circles) grown in the field under post-anthesis drought stress in WAR04FLD and for RTx7000 (open square) and four *Stg* QTL NILs (filled squares) grown in the field under post-anthesis drought stress in WAR05FLD. Data are averaged across replications and two plant densities (modified from Borrell *et al.*, 2014; with permission, Wiley).

Genotypic differences in individual grain mass under drought stress (WAR04FLD and WAR05FLD) were relatively small, suggesting that differences in grain number already reflected differences in assimilate availability, resulting in a significant correlation between grain number and yield (Fig. 3C). Individual *Stg* NILs exhibited higher (*P*<0.05) grain numbers than RTx7000 in four of the 14 treatment × *Stg* QTL combinations, with a trend for higher grain numbers in another eight of the combinations (Borrell *et al.*, 2014). In no combinations was grain number significantly less in the *Stg* NILs compared with RTx7000. In WAR04FLD, grain number per panicle was positively correlated with grain number m^–2^ under HD (*R* ^2^=0.91, *n*=4, *P*<0.01) and LD (*R* ^2^=0.76, *n*=4, *P*<0.05), suggesting that differences in grain number were primarily due to grain number per panicle rather than panicle number m^–2^. Furthermore, *Stg* NILs exhibited higher (*P*<0.05) individual grain masses than RTx7000 in three of the 14 treatment × *Stg* QTL combinations under drought, with a trend for higher grain masses in another seven of the combinations (Borrell *et al.*, 2014). In no combinations was individual grain mass significantly less in the *Stg* NILs compared with RTx7000.

The contribution of *Stg* loci to higher grain yield appears to be due to the extension of the photosynthetically active phase of the leaf and also possibly to higher photosynthetic rates. While the extension of green leaf area during grain filling is the key mechanism, the *Stg1* and *Stg4* NILs also exhibit higher specific leaf nitrogen at anthesis compared with RTx7000 (Harris *et al.*, 2007), suggesting that the photosynthetic rate may also be higher in these lines.

While the yield advantage of the NILs in these experiments is encouraging, it demonstrates the positive effect of the *Stg* introgressions on grain yield in only a single genetic background across four managed environments. To evaluate the value of stay-green across multiple genetic backgrounds and environments, Jordan *et al.* (2012) used data from the Queensland Government’s sorghum breeding programme to analyse the relationship between stay-green and yield from breeding trials that sampled 1668 unique hybrid combinations and 23 environments that ranged in mean yields from 2.3 to 10.5 t ha^–1^. While the majority of associations were positive in environments with yields below 6 t ha^–1^ (Fig. 2C), there was a trend towards a greater proportion of negative associations if trial mean yield increased above 8 t ha^–1^. However, the effectiveness of *Stg* QTLs depended on the genetic background, as the slope of the linear regression at a given grain yield differed consistently across male parents (Jordan *et al.*, 2012). Similar context dependencies have been shown in other studies (Kassahun *et al.*, 2010; Vadez *et al.*, 2011) that assessed multiple, rather than individual, *Stg* QTLs.

There was no consistent yield cost associated with the *Stg* QTLs in the irrigated control of WAR05FLD (Borrell *et al.*, 2014), supporting earlier studies showing that little or no yield penalty is associated with the BTx642 source of stay-green under high-yielding conditions (Borrell *et al.*, 2000b; Jordan *et al.*, 2012). Although biomass accumulation is radiation limited under well-watered conditions, the reduced leaf area index of *Stg* QTLs was still high enough to have minimal impact on intercepted radiation (Lafarge and Hammer, 2002).

Sorghum yields in Australia’s northern grain belt are currently about 2.5 t ha^–1^, and significantly less in central-western India and sub-Saharan Africa. Since grain yield of sorghum is likely to be affected by post-anthesis drought stress in rain-fed farming systems of northeastern Australia (Chapman *et al.*, 2002), India’s western-central monsoon region (DeLacy *et al.*, 2010), the southern USA (Rosenow *et al.*, 1983; Bandaru *et al.*, 2006), and sub-Saharan Africa (Dingkuhn *et al.*, 2006; Kouressy *et al.*, 2008; Abdulai *et al.*, 2012; Bhosale *et al.*, 2012), selection for stay-green in elite sorghum hybrids should have the potential to increase yield, profitability, and sustainability for farmers in rain-fed environments worldwide, without major costs during wetter years.

### Increased grain yield of *Stg* loci linked to their reduced canopy size at flowering

Reduced canopy size has been linked to increased grain yield under post-anthesis drought stress (van Oosterom *et al.*, 2011; Borrell *et al.*, 2014). Introgression of *Stg* QTLs into cereals will only benefit grain growers if changes in leaf-area dynamics associated with these QTLs do increase grain yield in environments characterized by post-anthesis drought (Fig. 1). At both WAR04FLD and WAR05FLD, *Stg* NILs consistently had lower green leaf area at anthesis (GLAA) under post-anthesis drought stress than RTx7000 (Fig 3A). In both experiments, biomass accumulation during the grain filling period (bioGFP) was significantly negatively correlated with GLAA (*R* ^2^=0.94, *n*=4, *P*<0.05 for WAR04FLD; *R* ^2^=0.83, *n*=5, *P*<0.05 for WAR05FLD; Fig. 3A). Under drought stress, when biomass production is a function of water availability, this negative relationship likely represents increased water availability during grain filling for the *Stg* NILs compared with RTx7000. In that case, genotypic differences in bioGFP likely reflect differences in the crop (panicle) growth rates around anthesis, which in turn determine grain number (van Oosterom and Hammer, 2008). Consistent with this, a positive correlation between bioGFP and grain number existed (Fig. 3B). Genotypic differences in grain number, in turn, explained most of the differences in grain yield (Fig. 3C). The increased grain yield of *Stg* QTLs under post-anthesis drought stress could thus be largely explained as an emergent consequence of pre-anthesis QTL effects on leaf-area dynamics.

### *Stg* loci decrease canopy size via reduced tillering and smaller upper leaves

**Fig. 4.**
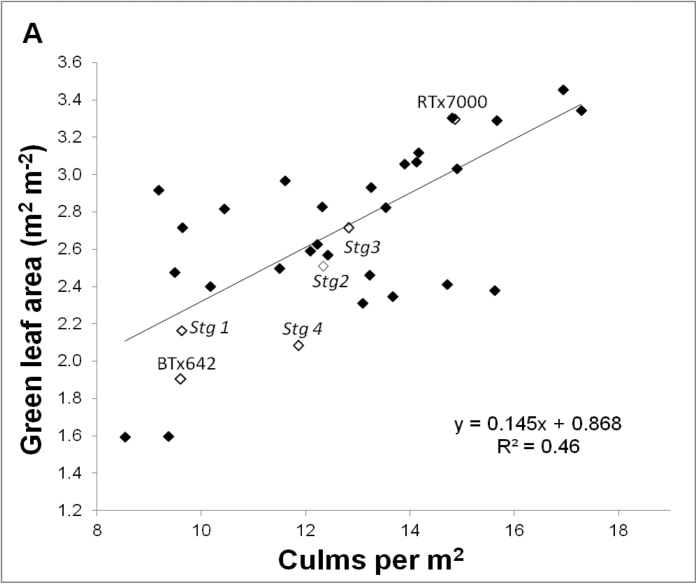

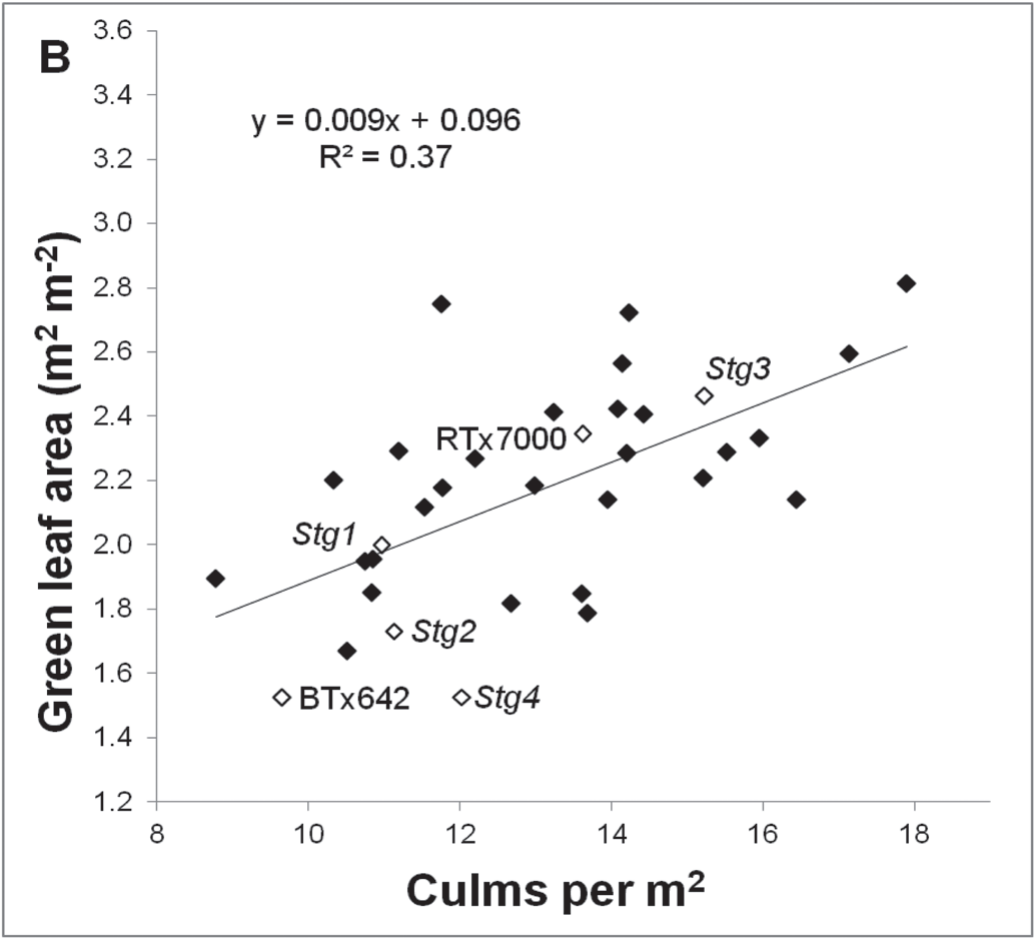

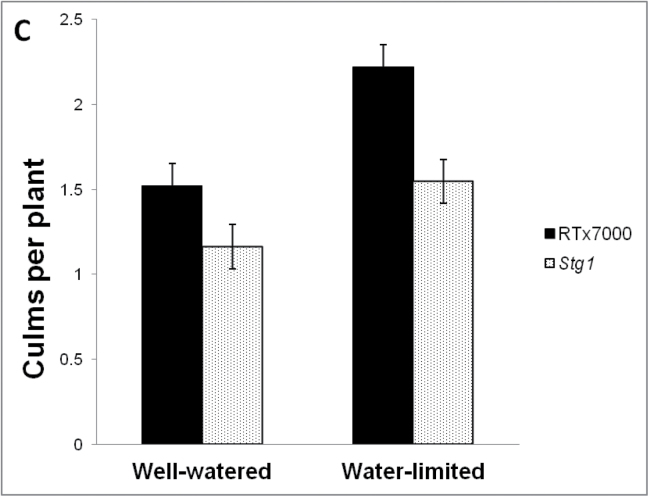

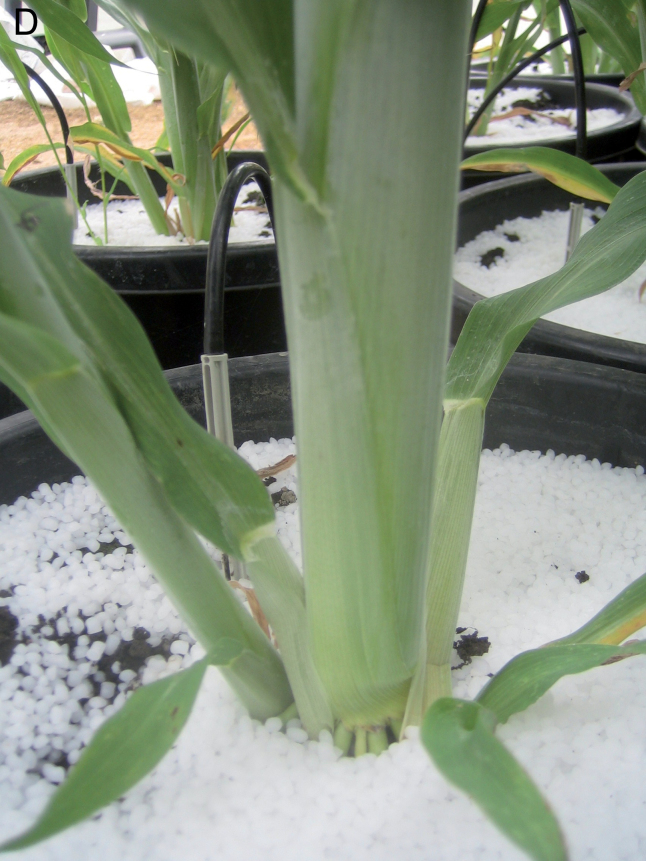

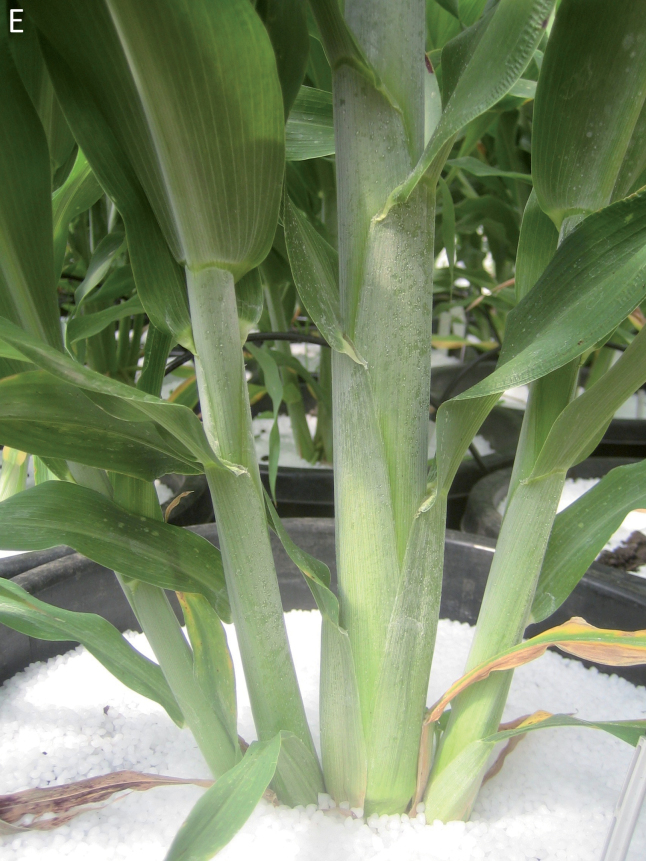
Reduced tillering by *Stg* QTLs is a constitutive trait. (A, B) Green leaf area at anthesis as a function of culm number m^–2^ in a set of 34 near-isogenic lines containing single or multiple introgressions of *Stg1*, *Stg2*, *Stg3*, and *Stg4* QTLs in a RTx7000 background grown under well-watered (A) and water-limited (B) conditions at BIL02FLD; the parental lines (RTx7000 and BTx642) and NILs containing only single introgressions of *Stg1*, *Stg2*, *Stg3*, and *Stg4* are highlighted. (C) Comparison of culms per plant between RTx7000 and the *Stg1* NIL under well-watered and water-limited conditions in WAR06FLD; data are mean±2SE. (D, E) The low-tillering phenotype exhibited by the *Stg1* NIL (D) relative to RTx7000 (E) in a pot experiment (WAR10POT) (this figure is available in colour at *JXB* online).

In general, introgressing the various stay-green QTLs into RTx7000 decreased the size of the canopy at anthesis by reducing (*P*<0.001) the number of culms m^–2^ under well-watered and water-limited conditions. Fig. 4A and B shows results for BIL02FLD, but similar results were obtained in other experiments. The overall ranking of tillering in BIL02FLD was *Stg1* < BTx642 < *Stg4* < *Stg2* < *Stg3* < RTx7000. In another field experiment under a rain-out shelter (WAR06FLD), introgressing the *Stg1* region alone into RTx7000 significantly (*P*<0.05) reduced culms m^–2^ compared with RTx7000 under both well-watered and water-limited conditions (Fig. 4C). Fig. 4D shows the low-tillering phenotype exhibited by the *Stg1* NIL relative to RTx7000 (Fig. 4E) in a pot experiment (WAR10POT). Low tillering of *Stg* isolines was significantly negatively correlated (*R* ^2^=0.97, *n*=5, *P*<0.01) with the larger size of leaves 2–9 relative to RTx7000 when evaluated across multiple experiments (Borrell *et al.*, 2014), suggesting that it was a consequence of the competition for limited carbon resources with larger developing leaves (Kim *et al.*, 2010; Alam *et al.*, 2014). Such a causal mechanism indicates that reduced tillering due to the *Stg* loci is a constitutive trait.

**Fig. 5.**
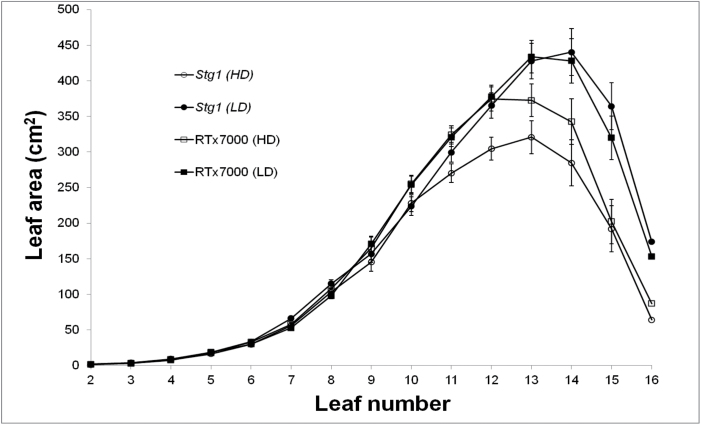
*Stg1* reduces the size of upper leaves under water deficit. Leaf size distributions of RTx7000 grown under high (open squares) and low (filled squares) densities and *Stg1* grown under high (open circles) and low (filled circles) densities in the WAR06FLD rain-out shelter study.

Individual *Stg* QTLs can also constrain GLAA by limiting the cumulative size of the upper leaves (Fig. 1; L10+), as a strong positive correlation (*R* ^2^=0.81 *n*=5, *P*<0.05) between the two traits has been reported (Borrell *et al.*, 2014). Evidence from six environments (Borrell *et al.*, 2014) showed that *Stg4* constrained the cumulative size of L10+ the most and *Stg1* the least. In WAR06FLD, where two levels of water deficit were generated by two levels of crop density, the leaf size distribution pattern of *Stg1* was similar to that of RTx7000 in the milder water deficit (LD), yet leaves were significantly (*P*<0.05) smaller in *Stg1* (up to 18% smaller) under the more severe water deficit generated by the HD treatment, indicating an adaptive response by *Stg1* plants to increasing water deficit (Fig. 5). Combined with the fact that upper leaves (L10+) elongate after tiller appearance has ceased, this suggests that the effects of *Stg* QTLs on GLAA via reduced size of L10+ operated through different mechanisms than the effects on GLAA via increased size of L2–9.

In summary, canopy development was largely regulated by two mechanisms in plants containing the *Stg* QTLs (Fig. 1): (1) reduced tillering in response to larger lower leaves (L2–9); and (2) smaller size of upper leaves (L10+). This implies major developmental changes around L9 and L10 (Borrell *et al.*, 2014). The first mechanism is likely to be most advantageous in low-density environments where tillering potential is high, whereas the second mechanism will likely dominate in high-density environments where tillering potential is low. Combined, these two mechanisms provide crop plants with considerable plasticity to modify canopy development in response to the severity of water limitation.

### Decrease in canopy size shifts crop water use from pre- to post-flowering

The extent of the effect of *Stg* QTLs on canopy development and grain yield varies with environmental and management conditions experienced by the crop prior to flowering (Fig. 3). This was illustrated by the experiment effect on the association between GLAA and bioGFP (Fig. 3A). One key physiological mechanism by which stay-green confers drought adaptation under terminal water deficit is to conserve soil water before anthesis for utilization during grain filling. Small increases in water use during grain filling can significantly impact grain yield, and simulation studies have found that 1mm of additional water transpired during grain filling could increase grain yield by about 30kg ha^–1^ in sorghum (Hammer, 2006) and by more than 55kg ha^−1^ in wheat (Manschadi *et al.*, 2006). These yield increases are supported by field experiments for wheat (Kirkegaard *et al.*, 2007) and are mainly due to the fact that water extracted late in the season is not required to build more structural crop biomass and is utilized predominantly for grain growth (Richards *et al.*, 2010). A critical aspect of any benefit from *Stg* QTLs on grain yield under drought is therefore the interaction of reduced canopy size and reduced crop water use at anthesis with environmental conditions, which determines water availability during grain filling (Fig. 1).

**Fig. 6.**
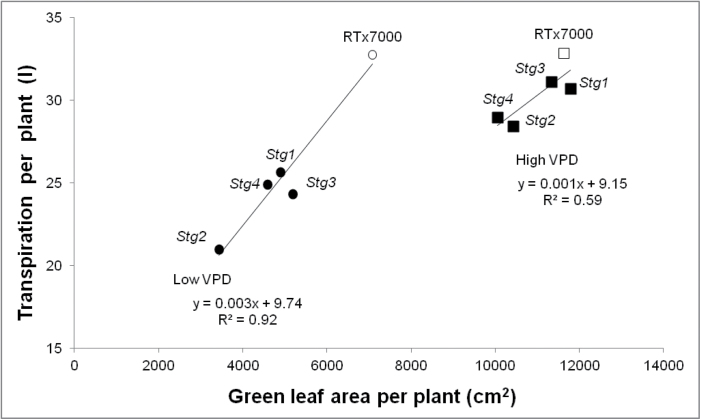
*Stg* QTLs reduce green leaf area and transpiration at anthesis. Transpiration per plant as a function of green leaf area m^–2^ at anthesis for RTx7000 (open circle) and four *Stg* NILs (filled circles) grown under low vapour pressure deficit (VPD) and RTx7000 (open square) and four *Stg* NILs (filled squares) grown under high VPD.

**Fig. 7.**
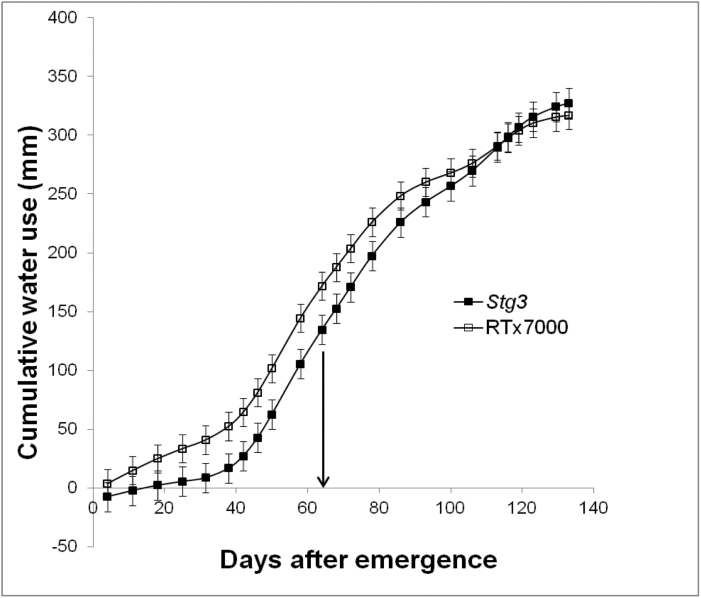
*Stg3* uses less water than RTx7000 before anthesis and more water after anthesis. The temporal pattern of cumulative crop water use for RTx7000 (open squares) and *Stg3* (filled squares) grown under the low-water high-density treatment in the WAR05FLD rain-out shelter study. The arrow marks anthesis.

A positive correlation between canopy size and water use at anthesis was observed under low vapour pressure deficit in WAR06LYS (*R* ^2^=0.92, *n*=5, *P*<0.01; Fig. 6), where canopy size at anthesis was significantly (*P*<0.05) less in all *Stg* NILs compared with RTx7000 and plant water use was significantly (*P*<0.05) less in *Stg2*, *Stg3*, and *Stg4*. Similar trends were observed under high vapour pressure deficit in WAR07LYS, although differences were not significant (Fig. 6). Importantly, these results were reproduced under field conditions in the rain-out shelter (LWHD treatment) at WAR05FLD, where crop water use in *Stg1*, *Stg3*, and *Stg4* was significantly (*P*<0.05) less than RTx7000 prior to anthesis, but significantly (*P*<0.05) more in *Stg1*, *Stg2*, and *Stg3* post-anthesis. For example, *Stg3* saved 37mm before anthesis compared to RTx7000 (135 versus 172mm; Fig. 7), which accounted for most of the additional 47mm used after anthesis (192 versus 145mm). Similar results were obtained for *Stg1*, which used significantly (*P*<0.05) less water than RTx7000 before anthesis (139 versus 172mm), but used significantly (*P*<0.05) more water after anthesis (197 versus 145mm). Crop water use during grain filling was positively correlated with grain yield in WAR05FLD across the LWHD and LWLD treatments (*R* ^2^=0.74, *n*=4, *P*<0.05), with the *Stg1* NIL using more (*P*<0.05) water and producing more (*P*<0.05) grain than RTx7000 under both densities (Borrell *et al.*, 2014). The slope of the relationship (5g m^–2^ mm^–1^ = 50kg ha^–1^ mm^–1^) was comparable to the 30kg ha^–1^ mm^–1^ reported by Hammer (2006) for sorghum and the 55kg ha^−1^ mm^–1^ reported by Manschadi *et al.* (2006) for wheat (both simulation studies). Therefore, the increased grain yield of *Stg* QTLs compared to RTx7000 under post-anthesis drought stress could at least partly be explained as a consequence of increased post-anthesis water use in response to reduced canopy size.

### *Stg* loci modify transpiration per unit leaf area via stomatal index

**Fig. 8.**
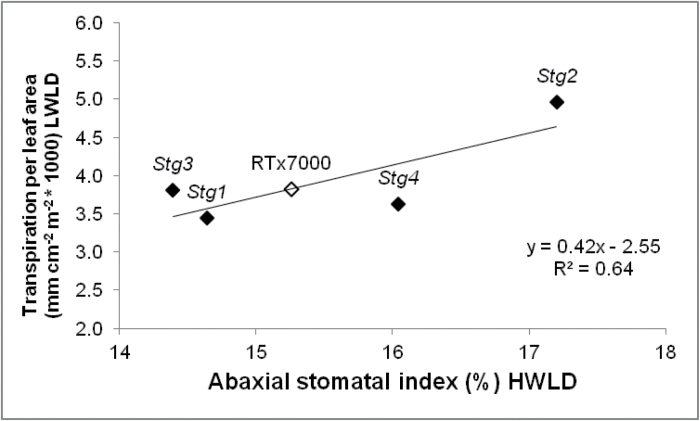
*Stg* QTLs modify transpiration per unit leaf area via abaxial stomatal index. The positive correlation between abaxial stomatal index (%) under high-water low-density (HWLD) conditions and transpiration per leaf area (mm cm^–2^ m^–2^ × 1000) under low-water low-density conditions (LWLD) for RTx7000 (open diamond) and the *Stg* NILs (closed diamonds) in the WAR05FLD rain-out shelter study.

Although the *Stg* loci reduced water use before flowering mainly by reducing transpirational leaf area, there is some evidence that the *Stg* loci also modified transpiration per unit leaf area (Fig. 8). In WAR05FLD, the abaxial SI under HWLD of Leaf 10 was significantly positively correlated (*R* ^2^=0.64, *n*=5, *P*<0.10) with average transpiration per unit leaf area during the pre-anthesis period measured in the adjoining LWLD treatment (Fig. 8). This suggests that the number of stomata could be a key determinant of transpiration per unit leaf area, which is a canopy-level measure of conductance. The data also indicates that particular *Stg* loci can either increase or decrease the SI and transpiration per unit leaf area, relative to RTx7000. The relatively low transpiration rate of *Stg4* in Fig. 8 was a consequence of its relatively high abaxial SI being offset by its small leaf size (Fig. 4), resulting in a relatively low transpiration per unit leaf area. Hence, *Stg* loci have an impact on the demand for water by regulating transpiration via at least two mechanisms: leaf area and transpiration per unit leaf area. This provides the crop with multiple pathways to conserve water.

### *Stg* loci modify root architecture and spatial water extraction patterns

**Fig. 9.**
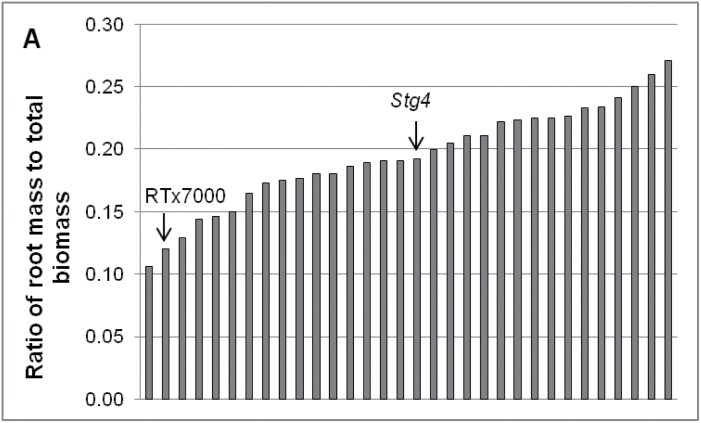

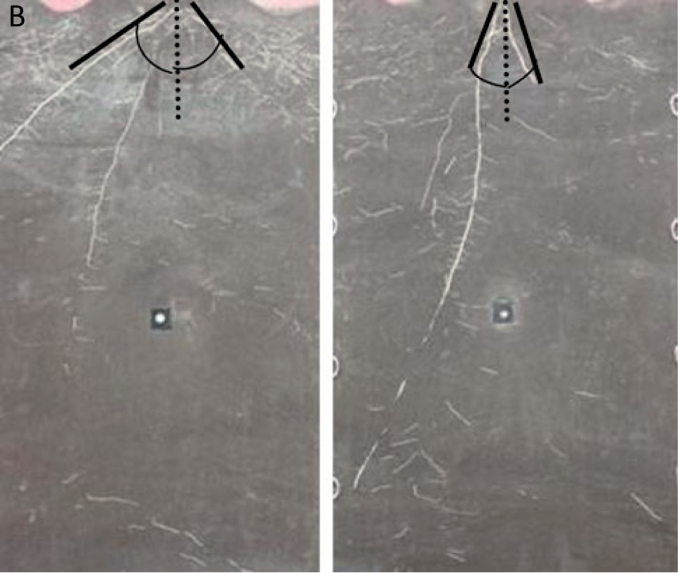

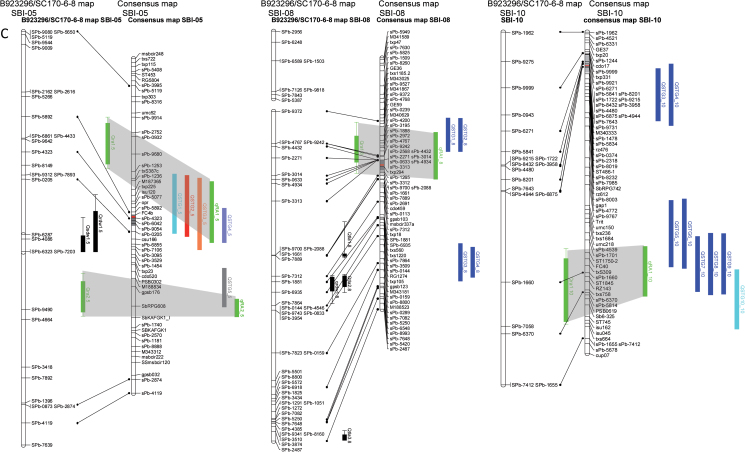
*Stg* QTLs affect root architecture. (A) Genetic variation for root harvest index in a *Stg4* fine-mapping population when harvested at the 5-leaf stage (WAR08POT). The parent of the population, RTx7000, and the *Stg4* NIL are indicated. (B) Nodal roots, visible on the glass surface of root chambers, for the parents of the RIL mapping population: SC170-6–8 (wide angle, left panel) and B923296 (narrow angle, right panel). Thick solid lines indicate first flush of nodal roots, dotted lines indicate the vertical plane, and arcs indicate the estimated root angle (from Mace *et al.*, 2012; with kind permission from Springer Science and Business Media: Springer and Oxford University Press, *Theoretical and Applied Genetics*, 124, 2012, 97–109, QTL for nodal root angle in sorghum (*Sorghum bicolor* L. Moench) co-locate with QTL for traits associated with drought adaptation, Mace E, Singh V, van Oosterom E, Hammer G, Hunt C, Jordan D, from Fig. 1). (C) Projection of the root angle QTLs onto the sorghum consensus map and comparison with stay-green QTLs identified in previous studies (from Mace *et al.*, 2012; with kind permission from Springer Science and Business Media: Springer and Oxford University Press, *Theoretical and Applied Genetics*, 124, 2012, 97–109, QTL for nodal root angle in sorghum (*Sorghum bicolor* L. Moench) co-locate with QTL for traits associated with drought adaptation, Mace E, Singh V, van Oosterom E, Hammer G, Hunt C, Jordan D, from Fig. 4); colour-coded as follows: light blue, Crasta *et al.* (1999; green, Feltus *et al.* (2006); dark blue, Haussmann *et al.* (2002); purple, Kebede *et al.* (2001); grey, Srinivas *et al.* (2009); orange, Subudhi *et al.* (2000); red, Xu *et al.* (2000).

The increased post-anthesis water use of *Stg* QTLs compared to RTx7000 was not solely a consequence of changes in temporal water-use patterns, as it more than compensated for the reduced pre-anthesis water use, resulting in increased total water use (Fig. 7). In the LWHD treatment of WAR05FLD, the *Stg1* QTL used 19mm more water than RTx7000 and the *Stg3* QTL 10mm more water (Fig. 7). As water uptake under drought stress is supply limited, these results indicate that the *Stg* QTLs could access more water than RTx7000. Increased access to water can be achieved by either better water extraction from the soil that is explored by the roots or increasing the soil volume explored by the roots through deeper rooting or greater lateral spread (Fig. 1; Manschadi *et al.*, 2006). There is some evidence that *Stg* QTLs could modify root architecture in sorghum. A *Stg4* fine-mapping population varied in biomass partitioning between root and shoot when harvested at the 5-leaf stage in WAR08POT (Fig. 9A). While genotypic differences were not significant (*P*>0.05), there was a trend for greater allocation to roots in the *Stg4* NIL (0.19) compared with RTx7000 (0.12).

In another study, Mace *et al.* (2012) mapped QTLs for nodal root angle in sorghum at the 6-leaf stage and evaluated the relevance of the trait for improving drought adaptation via marker-assisted selection. They assessed a subset of 141 F6 RILs that were developed by single seed descent from a cross between inbred lines B923296 (Fig. 9B, narrow angle relative to a vertical plane) and SC170-6–8 (wide angle). B923296 is a highly stay-green line containing the BTx642 source of stay-green. All four nodal root angle QTLs in sorghum identified by Mace *et al.* (2012) colocated with previously identified QTLs for stay-green (Fig. 9C; adapted from Mace *et al.*, 2012). In fact, the peak LOD location of all four QTLs occurred within a stay-green QTL region. Importantly, qRA1_5 colocated with the *Stg4* QTL, with the peak location of qRA1_5 occurring within four previously identified QTLs for stay-green (Crasta *et al.*, 1999; Subudhi *et al.*, 2000; Xu *et al.*, 2000; Kebede *et al.*, 2001). Therefore the trend for increased root HI exhibited by the *Stg4* NIL in WAR08POT is likely to contribute to the stay-green phenotype in this NIL.

Genotypic differences in nodal root angle can affect spatial water-use patterns of mature plants. Singh *et al.* (2012) observed a trend that a genotype with a narrow root angle (vertical root system) had relatively more root length directly below the plant than a genotype with wide root angle (horizontal root system), which produced more lateral roots, resulting in more lateral water uptake. Although differences in relative water extraction were not significant, these preliminary results do support the hypothesis that a narrow root angle may increase water accessibility in deep soils under higher plant density, whereas a wide root angle may increase water accessibility under lower density (e.g. skip row) management systems. These results support the inclusion of root angle as a selection criterion in sorghum breeding programmes.

## Conclusions

Stay-green is an important drought-adaptation trait in cereals. *Stg* loci reduce canopy size at flowering by modifying tillering, leaf number, and leaf size. Smaller canopy size at flowering reduces pre-anthesis water use, which under post-flowering water stress increases water availability during grain filling and thus grain yield. There is also some evidence that *Stg* loci have an impact on root architecture, which is likely linked to the increased water accessibility during grain filling under field conditions. *Stg* loci can also modify leaf anatomy, affecting parameters such as abaxial SI. The stay-green phenotype is thus the emergent consequence of the interaction between *Stg* loci that regulate largely constitutive traits related to plant size, and hence water demand by the crop, and the environment that regulates water supply by the soil. It is anticipated that the genes that underpin *Stg* QTLs could be modulated in other major cereal species (wheat, maize, and rice) to enhance their drought adaptation in localities worldwide where water is limiting after flowering.
